# Characterization of Early Age Curing and Shrinkage of Metakaolin-Based Inorganic Binders with Different Rheological Behavior by Fiber Bragg Grating Sensors

**DOI:** 10.3390/ma11010010

**Published:** 2017-12-22

**Authors:** Giovanna Palumbo, Agostino Iadicicco, Francesco Messina, Claudio Ferone, Stefania Campopiano, Raffaele Cioffi, Francesco Colangelo

**Affiliations:** 1 Department of Engineering, University of Naples “Parthenope”, Centro Direzionale Isola C4, 80143 Naples, Italy; giovanna.palumbo@uniparthenope.it (G.P.); agostino.iadicicco@uniparthenope.it (A.I.); francesco.messina@uniparthenope.it (F.M.); claudio.ferone@uniparthenope.it (C.F.); stefania.campopiano@uniparthenope.it (S.C.); raffaele.cioffi@uniparthenope.it (R.C.); 2 Research Group Naples Parthenope, National Interuniversity Consortium of Materials Science and Technology (INSTM), Via Giuseppe Giusti, 9, 50121 Florence, Italy

**Keywords:** geopolymer, early age properties, shrinkage, metakaolin, rheology, fiber optic sensors, structural health monitoring

## Abstract

This paper reports results related to early age temperature and shrinkage measurements by means fiber Bragg gratings (FBGs), which were embedded in geopolymer matrices. The sensors were properly packaged in order to discriminate between different shrinkage behavior and temperature development. Geopolymer systems based on metakaolin were investigated, which dealt with different commercial aluminosilicate precursors and siliceous filler contents. The proposed measuring system will allow us to control, in a very accurate way, the early age phases of the binding systems made by metakaolin geopolymer. A series of experiments were conducted on different compositions; moreover, rheological issues related to the proposed experimental method were also assessed.

## 1. Introduction

Research on structural health monitoring has increased in a significant way over recent decades. Particularly, this topic is relevant to civil and structural engineering applications. In civil engineering, ageing of infrastructures represents a major issue, due to both structural safety and economic loss owing to maintenance operations. These reasons determined several research initiatives towards the development and design of new sensing technologies for multipurpose applications, particularly regarding the monitoring of existing structures. 

The durability of a civil infrastructure is significantly determined by the quality of the materials. Regarding structural materials, real time monitoring is a strategic process that is used for the highly accurate engineering design of composite components, such as fiber reinforced polymers, high-performance concrete, etc. Recently, several non-destructive techniques for real time monitoring of significant engineering parameters such as setting time definition [1,2,3], mechanical properties development, or loss [4,5,6], etc., have been proposed in literature. Real time monitoring is very useful for the characterization of early age properties of structural material. This process is very relevant to obtaining highly durable and high-performance materials with respect to aggressive exposure classes, since thermal and shrinkage phenomena can determine a high risk of early age cracking patterns, which are highly detrimental to service life.

The most consumed binder in the construction industry is ordinary Portland cement (OPC), and its main composite is concrete. The manufacturing of OPC requires the burning of large quantities of fuel and the decomposition of limestone, leading to significant emissions of carbon dioxide, which are further increased by other components (aggregates, fibers, chemical admixtures, etc.) of concrete materials [7,8,9]. For this reason, during the last decade, major research efforts were directed towards finding new sustainable and environmentally friendly composites in order to replace conventional concrete, even if existing literature often neglects the contribution of OPC-based materials to CO_2_ storage.

Alkaline activation may represent an effective technology for reducing the environmental impact of binders realized by means of alkaline activation, which are usually referred to as alkali activated materials (AAM). AAM can be synthesized by means of the alkaline activation of several solid precursors such as coal fly ash [10,11,12], calcined clays [13,14,15], etc., allowing at the same time for the reduction of environmental impact with respect to clinker production and the achievement of valuable engineering properties, in terms of mechanical [16], environmental [17], and functional [18,19,20] performance. In the case of inorganic binders such as the cementitious ones, one of the main technological issues is represented by early age cracking, which is caused by internal and external factors that have mechanical, thermal, or hydraulic origins [21]. Due to the hydration process and external influences, different types of deformation and non-desirable stresses may appear [22,23]. These cracks could act as passages that favor an attack from harmful agents and quickly reduce the durability of the concrete [24,25]. Construction materials exhibiting early age cracking have low durability, show an overall deterioration due to aging, and need frequent maintenance and conservation operations, or even their integral replacement, increasing the consumption of raw materials and costs [26]. For these reasons, a proper knowledge of the early age properties represents a key parameter to: (i) assess the risk of early age cracking; and (ii) optimize the mix design. Shrinkage is the decrease in volume of concrete with time, or rather, the deformation measured in an unloaded and unrestrained specimen at constant temperature in laboratory conditions [27,28].

More recently, evidence of optical fiber technology as a solution to the direct temperature and strain measurement of early-age cement-based materials was reported, due to its high precision, high resolution, flexibility, insensibility to electromagnetic fields, and mechanical robustness [29]. Fiber optic sensors (FOSs) are small in size, lightweight, easy to embed, and low cost. Moreover, they have high temperature endurance, are able to perform in-situ sensing at multiple locations, can work in harsh conditions, and are chemically inert so they do not affect the properties of the material that they come into contact with [30,31]. Finally, FOSs can be developed to selectively detect, for example, variation in strain, temperature, corrosion, or crack formation [32]. 

Among FOSs, Fiber Bragg Gratings (FBGs) have been investigated as a sensing platform in several applications, ranging from aerospace to chemical and physical [32,33,34,35]. Particularly, FBGs have been successfully applied to civil engineering in order to monitor displacements and cracks and also to measure strain and temperature [29,36]. Whelan et al. developed a prototype system for on-line structural monitoring of the cathedral of Como (Italy), employing FBGs [37]. Tennyson et al. applied FBG’s technology to measure static and dynamic loads on bridges in Canada (ISIS—Intelligent Sensing for Innovative Structures) [38]. Other applications reported the use of FBGs to measure the behavior of different components of the tunnel [39] and to monitor crack in long pipelines and dams [40,41], or even to function as pressure sensors and monitor seismic activity in reservoir, oil, and gas pipelines, and well-drilling applications [42,43,44,45,46]. However, until now, few investigations dealing with early-age shrinkage using fiber-optic sensors were presented: these included the use of Fabry–Perot interferometers embedded in the concrete structure that was used to sense temperature changes during the hydration process [47], low-coherence interferometry [39], and packaged FBGs on cement paste [48,49,50]. Fiber Bragg Gratings, thanks to their advantageous features, represent an appealing solution to the problem of simultaneous measurement of temperature and strain in real-time during the drying phase of alkali-activated metakaolin (AAMK), which is employed easily in the case of small containers [51]. Moreover, FBGs can be used under extreme conditions (e.g., high strain and high temperature), allowing for a higher degree of interaction with the investigated material than other types of sensors.

In this paper, the study of early age shrinkage and temperature changes of AAMK samples, through a fiber Bragg grating system, was reported. Particularly, several FBGs were properly embedded in AAMK matrices in order to obtain real time measurement after pouring. The proposed system allows us to control the early age behavior of the binding systems made by metakaolin geopolymer while considering different aluminosilicate precursors and filler contents. Systems were also characterized in terms of setting time, rheological performance, and early age mechanical properties. Obtained data highlighted the highly reliable measuring performance of the developed experimental setup, which needs to be further optimized in order to be proposed for future technical standards. 

## 2. Experimental Program

### 2.1. Geopolymers Binding Systems: Design and Characterization

The composition of investigated binding systems is reported in Table 1. The research focuses on paste samples in order to maximize shrinkage effects determined by binder phase, since aggregates contained in concrete reduce the magnitude of shrinkage in a significant way. Furthermore, the presented experimental setup is not adequate for testing concrete samples due to reduced diameter of used vials (see Section 2.3), but it will be upscaled in subsequent experimental studies. Three different commercial metakaolin precursors were used provided by Kimia (Italy), Argeco (France), and Neuchem (Italy), respectively. 

The alkaline activating solution was prepared by using NaOH pellets (Baker, analytical *R* grade) and sodium silicate solution (*R* = 3.2) supplied by Prochin. A pure quartz powder provided by Sibelco (MILLISIL SA12, SiO_2_ 99 wt %; maximum particle diameter 63 μm; specific surface area 0.35 m^2^/g) was added as filler to the metakaolin and the activating solution. Alkaline activating solutions were prepared before mixing phases, which were realized in a Hobart mixer for 10 min. The fresh mixes were poured into a series of cylindrical polyethylene molds of size *d* × *h* = 3 cm × 6 cm for mechanical testing. The samples were stored in sealed conditions at 40 °C in a climatic chamber with the same conditions used for FBG measurements. The obtained specimens were subjected to compressive strength determination by using a 100 kN capacity Controls MCC8 testing machine. Compressive strength determinations were carried out at 2, 7, and 14 days in order to assess early age mechanical properties. 

Since there is a time interval needed for sample preparation and test starting of FBG measurements, setting time was measured by means of Vicat needle testing procedure. This measurement is needed to verify that the so-called dormant period of hardening process is not concluded before the preparation of experimental measurements with FBGs. Furthermore, since air bubble formation associated with sample preparation can affect FBG measurements, rheological characterization of binding systems was performed by means of V-funnel testing and dynamic minislump cone testing. For V-funnel testing, a plastic funnel with upper diameter, lower diameter, and height equal to 8.0, 2.5, and 7.0 cm, respectively, was used. V-funnel test measurements rely on the evaluation of emptying time of the funnel by the paste immediately after mixing, and it is helpful to assess viscosity relatively among the various investigated mixtures. Minislump cone test represents a simple procedure, which is found quite frequently in literature, since it allows for the making of both qualitative and quantitative observations of the fresh slurries. In this work, the geometry of the minislump cone was as follows: Top diameter equal to 5.0 cm; bottom diameter equal to 6.8 cm; height equal to 6.5 cm (corresponding to a paste volume equal to 179.1 cm^3^). Geopolymeric slurries were slowly poured in the cone placed on a horizontal plane and carefully compacted by means of a thin steel rod. Slump orthogonal diameters were measured, and the average diameter was used to calculate minislump area. In scientific literature related to cementitious composites, slump values are well-correlated with yield stress values. It is useful to qualitatively assess yield stress by performing rheological measurements, since this parameter is associated with the capability of the fresh mixture to entrain air. Minislump measurements were repeated at 0, 15, 30, 60, and 100 min in order to evaluate workability loss in the very early age by keeping the fresh slurries in controlled environment (*T* = 20 ± 2 °C; sealed container).

### 2.2. FBGs Principle of Operation

**Figure 1:**
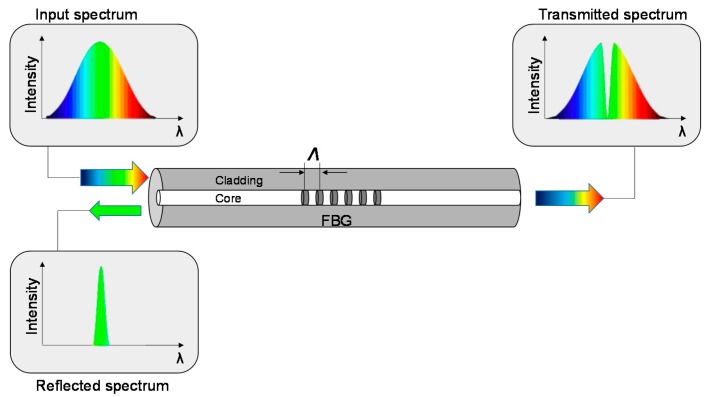
Schematic of fiber Bragg gratings (FBG) structure with spectral profile.

The Fiber Bragg Grating is an optical sensor inscribed within the core of a standard single-mode optical fiber with typical length of 1–20 mm. This device consists of a periodic modulation of the core refractive index along the length of fiber, obtained by exposing the fiber to ultraviolet light, and due to photosensitivity property of the doped silica glass fiber core (Figure 1). 

The refractive index modulation leads to the reflection of the light propagating along the fiber in a narrow range of wavelengths known as the Bragg wavelength, whereas the rest of the spectrum is transmitted. The Bragg wavelength is related to the grating period (Λ) and to the effective refractive index of the core propagation mode ($n_{eff}$) by:

(1) $$\lambda_{B}=2n_{eff}\Lambda ,$$

The effective refractive index of the core and the spatial periodicity of the grating are both affected by changes in strain (Δε) and temperature (ΔT). As a result, the Bragg wavelength changes its position, according to the following equation [52,53,54,55]:

(2) $$\Delta \lambda_{B}=2\left(\Lambda \frac{dn_{eff}}{dT}+n_{eff}\frac{d\Lambda }{dT}\right)\Delta T+2\left(\Lambda \frac{dn_{eff}}{d\varepsilon }+n_{eff}\frac{d\Lambda }{d\varepsilon }\right)\Delta \varepsilon ,$$

The first term on the right hand side of the Equation (2) is related to the effect of temperature. This term, in turn, depends upon the temperature-induced change in the effective refractive index (known as thermo-optic effect: $\frac{1}{n_{eff}}\frac{dn_{eff}}{dT}=\xi$) and in the thermal expansion of the grating ($\frac{1}{\Lambda }\frac{d\Lambda }{dT}=\alpha$).

The second term in Equation (2) is related to the effect of longitudinal strain applied to the optical fiber. It arises from the strain-induced change in the grating periodicity and from elasto-optical-induced change in the refractive index. 

Hence, it is possible to rewrite Equation (2), introducing the thermal sensitivity coefficient $S_{T}=\left(\xi +\alpha \right)$ and strain sensitivity coefficient$\;S_{\varepsilon }=\left(1-p_{e}\right)$, in which $p_{e}\;$is the effective photo-elastic coefficient. Consequently, the Bragg grating wavelength shift due to strain and temperature changes can be expressed as:

(3) $$\frac{\Delta \lambda_{B}}{\lambda_{B}}=S_{T}\Delta T+S_{\varepsilon }\Delta \varepsilon ,$$

In order to separate the two terms of Equation (3) into strain and temperature simultaneous measurements, it is a common practice to use two FBGs: one in a free and unstrained condition, so that it can only record temperature changes; the other recording both strain and temperature changes effects. The measure of the latter FBG will then be compensated by eliminating the temperature effects recorded by the former (which is strain-free).

### 2.3. Sensors Design

For the aim of this paper, several AAMK samples with different compositions (see Table 1) were prepared and poured into a cylindrical holder in which the early age shrinkage and temperature were measured. Two FBG sensors properly packaged were necessary for each AAMK sample to discriminate shrinkage and temperature behavior. The FBG temperature sensors were properly inserted in a metallic needle in order to protect the grating and create a strain-free condition [55]. 

**Figure 2:**
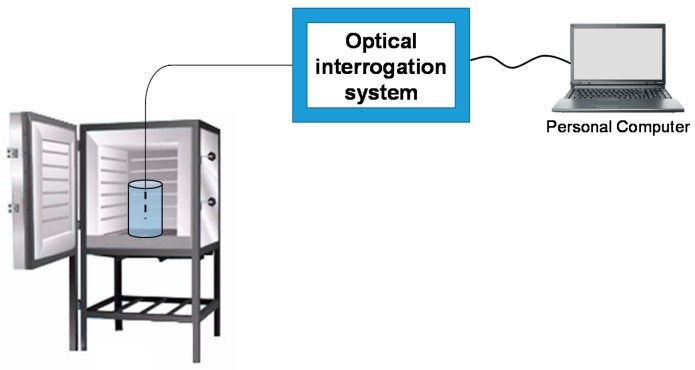
Schematic of: experimental measurement set-up (not in scale).

The sensors were fixed in cylindrical polyethylene molds of size *d* × *h* = 3 × 6 cm, in which fresh mixes were poured. A small hole was made at the bottom and on the lid of the mold to allow the passage of the optical fiber on both ends of the shrinkage sensor, whereas the temperature sensor was left hanging from the top cover. The samples were cured for fourteen days at 40 °C, keeping the lids closed to ensure 100% relative humidity. Finally, in addition to these sensors, we used a FBG positioned in the chamber, being strain-free and in proximity of the metakaolin samples as a temperature sensor. A schematic view of the experimental measurement set-up is shown in Figure 2. A HBM commercial optical interrogator was used to acquire the FBGs reflected spectrum (a BraggMeter FS22 from Fibersensing). In particular, it includes a tunable source in the range of 1500–1600 nm. The detector measures the Bragg wavelengths with a resolution of 1 pm with a minimum sampling frequency of 1 Sample/s; it is possible to detect temperature with a resolution of 0.1 °C and a strain with a resolution of 1 με. Moreover, an oven with temperature control up to 250 °C and temperature resolution of 1 °C was used.

## 3. Experimental Results and Discussion

**Figure 3:**
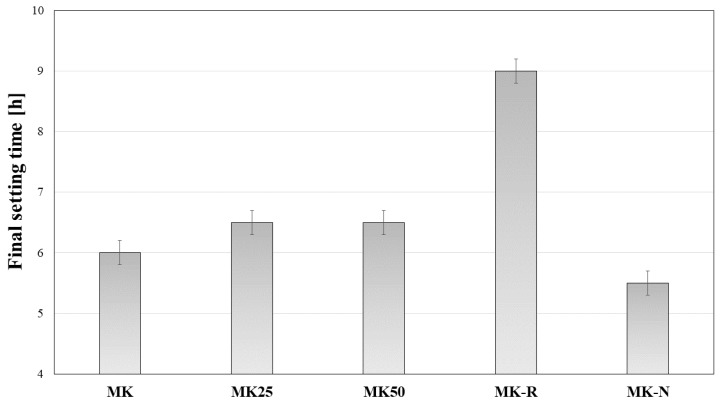
Setting time measurements.

The experimental program reports both characterization of materials and subsequent measurements by means of FBG sensors. After mixing phases, the geopolymeric slurries need to be placed in developed setup for FBG-based measurements. The time needed to prepare FBG measurements after first contact between solid precursors and alkaline activating solution can be estimated to be about 40 min. In this time frame, it is fundamental to assess setting performance associated with the initial phases of polycondensation reactions which are typical for AAMK systems. In Figure 3, results related to final setting time were reported. Dormant period represents the time interval characterized by the absence of hardening phenomena. In the dormant period, the material keeps the initial fluid/plastic state, and only minor rheological modifications occur. For all the investigated mixtures, final setting occurs in a time range between 5.5 and 9 h. These results, associated with following results on the observed rheological behavior, provide confirmation that time needed for FBG testing preparation (about 40 min) is reasonable with respect to polycondensation and hardening phases.

It must be pointed out that paste sample MK-R, i.e., the sample realized by using flash calcined metakaolin, provided a slower reactivity for the designed composition. This result is useful to support discussion of following measurements. In the other cases, namely for MK, MK25, MK50, and MK-N, results related to final setting are in a narrow range. Particularly, MK-N exhibited the fastest setting behavior, while filler content of MK25 and MK50 samples influenced, in a limited way, the setting kinetics of the reference mixture (MK). 

**Figure 4:**
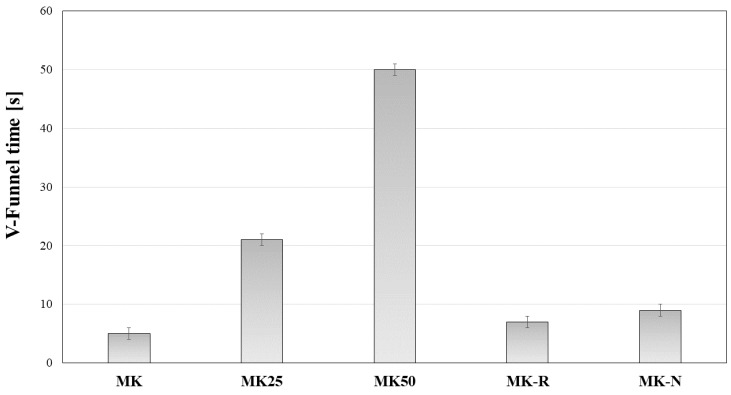
V-funnel time for AAMK binding systems.

**Figure 5:**
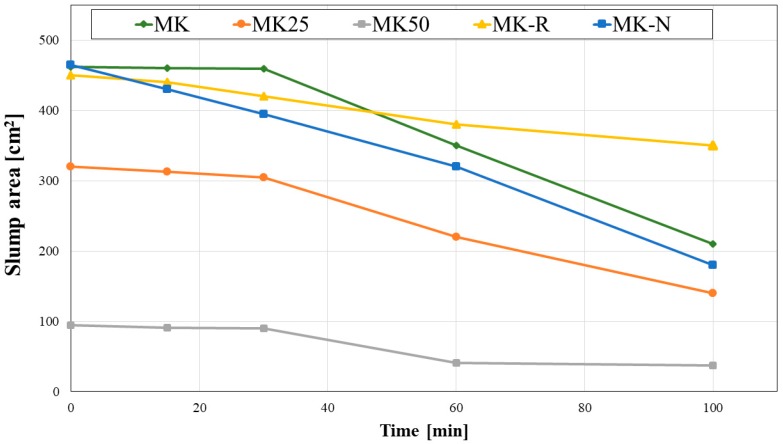
Slump flow dynamic measurements.

In Figure 4 and Figure 5, experimental results related to rheological measurements are reported. 

V-funnel time is empirically related to viscosity of slurries. These measurements express the rheological performance immediately after mixing phases. By means of these measurements, it is possible to evaluate the risk of bubble formation. In this case, results reported in Figure 5 clearly show that filler content represents a critical parameter for the increase of V-funnel time. Particularly, it is observed that 25% (respect to metakaolin mass) addition of quartz filler (MK25) determines an increase of V-funnel time by a factor of about 4 with respect to the reference mixture (MK). Further addition of quartz filler (MK50) causes an increase of V-funnel time by a factor of about 10. Hence, viscosity of slurries and associated risk of bubble formation is relevant for systems with higher filler content. During the phases of FBG testing preparation, this rheological information was confirmed by visual inspection of samples. In this regard, MK50 sample showed the formation of several air bubbles, which were observed through the transparent mold. 

In Figure 5, slump flow dynamic measurements are reported. MK, MK-R, and MK-N exhibit similar behavior, even if slight differences arise. Particularly, MK-N shows a faster loss of workability in the first hour after mixing. This result can be associated with faster kinetics of the solid aluminosilicate precursor used for MK-N synthesis. Furthermore, the faster reduction of slump flow for MK-N is in agreement with Vicat needle testing results, since MK-N final setting occurs after 5.5 h. Filler containing geopolymeric slurries, namely MK25 and MK50, presents significantly reduced initial slump area values, even if the general trend of dynamic slump evolution is similar to the one exhibited by MK sample. MK50 represents a critical system from a rheological point of view also in this case. 

**Figure 6:**
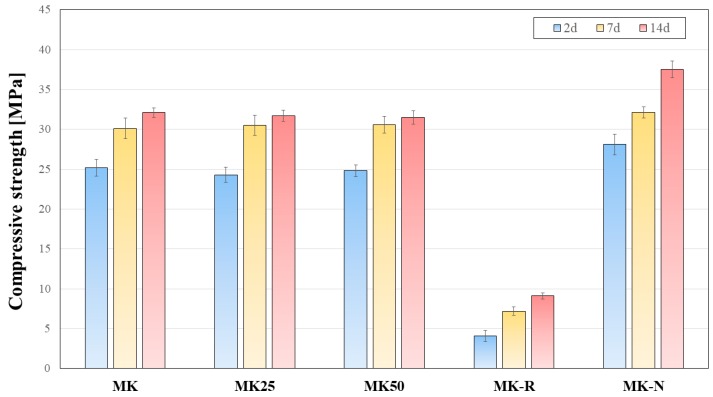
Compressive strength results for AAMK binding systems cured at 40 °C.

In Figure 6, the results related to early age mechanical properties development are reported. Geopolymeric binders realized by means of flash calcined metakaolin, i.e., MK-R, exhibited a poor mechanical performance that allowed only applications for civil purposes when high strength values were not strictly required, at least at early age. This result is in agreement with delayed setting observed in the case of Vicat test (see Figure 3). Filler content showed no significant influence on mechanical performance of AAMK binding systems. MK-N reported the highest compressive strength values at both very early (after 2 days curing) and early (after 7 and 14 days curing) age. It is fundamental to observe that very early mechanical properties development is quite relevant for AAMK systems, mainly considering MK, MK25, MK50, and MK-N systems. Particularly, unconfined compressive strength values after 2 days curing at 40 °C in sealed conditions are higher than 20 MPa for all four previously mentioned samples, reaching a maximum value of 28.1 ± 1.3 MPa for MK-N sample. Hence, the evaluation of shrinkage and thermal phenomena at very early age is fundamental because most of the engineering properties of these AAMK systems are developed immediately after final setting time. Other alkali activated systems such as fly ash-based ones present much slower reactivity and can be considered less critical with respect to early age cracking phenomena.

Experimental measurements focused on the temperature and strain recorded by FBG sensors during polycondensation process of the geopolymer materials. Particularly, the temperature and strain were calculated from the respective Bragg wavelength shifts during the process, whereas the strain was calculated starting from FBG shrinkage sensors, using the FBG temperature sensors for the compensation. 

**Figure 7:**
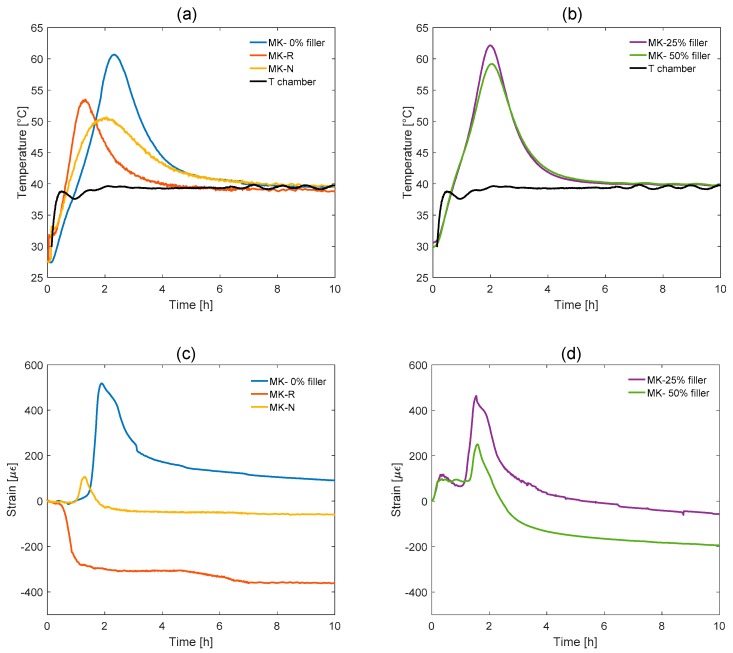
Temperature curves of the first ten hours of: (**a**) the first group of AAMK samples and (**b**) the second group of AAMK samples. Shrinkage curves of the first ten hours of (**c**) the first group of AAMK samples and (**d**) the second group of AAMK samples.

All the systems reach thermal equilibrium in a controlled oven at 40 °C after heat from reaction is decreased. This is the reason for setting curing conditions for AAMK pastes at 40 °C in sealed conditions in order to perform significant mechanical testing. In Figure 7, the temperature and shrinkage curves of the first ten hours of the AAMK samples are shown. Particularly, in Figure 7a, the temperature curve related to the first group of samples, i.e., MK, MK-N, and MK-R, is shown in order to highlight the influence of different aluminosilicate precursor. The three samples show different temperature peaks located at different time instants. In all cases, the thermal peak is relevant and accurately describes the typical kinetics of metakaolin-based alkali activated systems, with a significant associated thermal phenomenon. In Figure 7b, temperature changes of the second group of samples (MK25 and MK50) are reported in order to highlight the influence of filler content with respect to reference mixture (MK). In this case, we recorded the same behavior, in fact the maximum temperature occurred at the same time (after about two hours from the start of the test). The values of the maximum temperature recorded in the samples tested are reported in Table 2. 

In Figure 7c,d, the shrinkage curves of the first ten hours of the two groups of AAMK are shown. As shown in Figure 7c and also in the summary Table 2, for the first group, positive strain peaks (corresponding to a tensile stress) are observed for the samples MK-0% filler and MK-N, while the MK-N sample does not present any peak of positive strain. Observing the strain behavior for the second group (Figure 7d), as already noted from the temperature monitoring, the samples show similar strain behavior, with initial positive strain at the same time (the values are given in Table 2).

**Figure 8:**
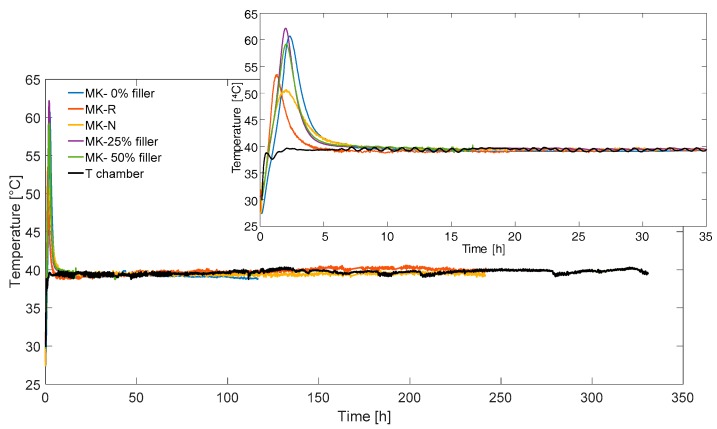
Temperature curves for different AAMK samples obtained by means of FBG. A zoom of the first thirty-five hours is in the insert.

In Figure 8, we reported the temperature curves of different AAMK samples recorded during the whole experiment, which lasted 330 h (about fourteen days). The measure for each sample was interrupted, as the material reached its stability. Thermal peak values are slightly different for the two groups of samples. All samples reached the thermal equilibrium with oven after a certain time interval.

**Figure 9:**
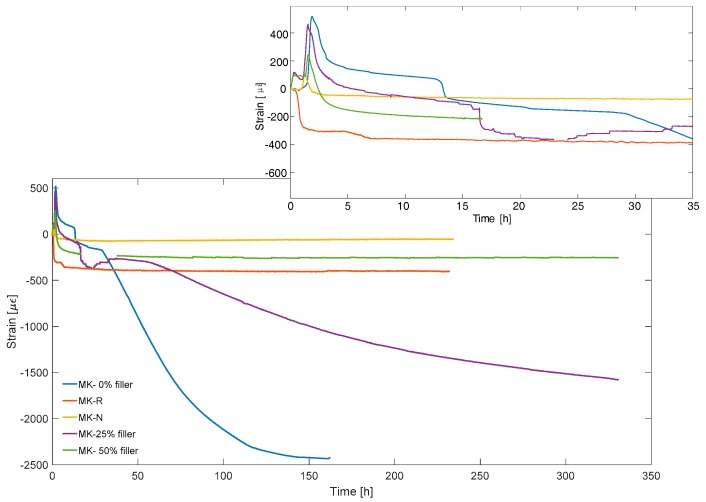
Shrinkage curves for different AAMK samples obtained by means of FBG. A zoom of the first thirty-five hours is in the insert.

Figure 9 shows the shrinkage curves recorded by FBGs. The early age crack may occur when mechanical properties of the material are not completely developed, so in this step the magnitude of shrinkage is relevant. Indeed, in the case of MK system, the risk of early age cracking in real scale applications could be high.

## 4. Conclusions

The FBG-based experimental setup presents critical issues with respect to rheological properties of investigated systems, since materials cannot be vibrated easily. Hence, air bubbles formation must be avoided in order to obtain proper measurements. This aspect is critical for several experimental setups used for the measurement of early age properties. Dealing with shrinkage, an example is given by the setup used in the so-called corrugated tube method, which represents the international standard for autogenous shrinkage measurement in the case of cementitious mortar [56,57]. Hence, the materials investigated were also characterized in terms of rheological properties. The system MK50, i.e., a composite binder realized by means of 50% (with respect to metakaolin mass) filler addition, was revealed to be a limit system with respect to rheological properties. A further reduction in viscosity and an increase in yield stress (considering, for instance, a Bingham model) determine a significant reduction in test significance. Particularly, the experimental program highlighted the following results:

- Mix design optimization was realized on a different scale with respect to a typical concrete scale used for inorganic binder-based composites for both structural and non-structural applications, determining the influence of both different solid aluminosilicate precursors (namely commercial metakaolin powders) and different filler contents respect to a reference binder mixture (i.e., MK).
- In one case, i.e., in the case of MK-R, which is the geopolymeric binder obtained using flash calcined metakaolin, measurements with FBG sensors provided results with minor significance, probably associated with the very low reactivity of the designed system that was verified by observing the delayed setting and poorly developed mechanical performance at an early age. From visual inspection of the samples, MK-R exhibited also a high degree of efflorescence on the surface, confirming that this solid precursor needs a specific mix design to be performed in subsequent research studies.
- Rheological limits for the application of the developed FBG-based experimental setup were found in the system MK50, for which the formation of air bubbles became critical due to higher viscosity and yield stress with respect to other mixtures investigated in the present study. The assessment was carried out on a qualitative basis, since existing literature confirmed the empirical correlation of slump and V-funnel time with yield stress and viscosity, respectively [58,59].
- FBG-based real-time monitoring of temperature and shrinkage in simultaneous AAMK systems was successfully achieved with a high degree of accuracy, particularly with a temperature resolution of 0.1 °C and a strain resolution of 1 με.
- FBG-based real-time monitoring of early age properties can be used and further optimized for the improvement of the existing standards for the experimental characterization of both traditional and innovative binders.

## Figures and Tables

**Table 1:** Mix design composition of alkali-activated metakaolin (AAMK) samples.

(g/100 g MK)	MK	MK25	MK50	MK-R	MK-N
Kimia MK	100	100	100	-	-
Argeco MK	-	-	-	100	-
Neuchem MK	-	-	-	-	100
Sodium silicate	120	120	120	120	120
NaOH pellets	20	20	20	20	20
Filler	-	25	50	-	-

**Table 2:** Temperature and shrinkage values recorded.

Sample	Temperature		Strain	
	Time [min]	Value [°C]	Time [min]	Value [με]
**Group 1**				
MK-0% filler	138	60.7	132	+517.9
MK-N	120	50.6	78	+106.5
MK-R	78	53.2	-	-
**Group 2**				
MK-25% filler	120	62.1	73	+462.6
MK-50% filler	120	59.1	73	+249.9
